# Shedding light on the prefrontal correlates of mental workload in simulated driving: a functional near-infrared spectroscopy study

**DOI:** 10.1038/s41598-020-80477-w

**Published:** 2021-01-12

**Authors:** Christoph F. Geissler, Jörn Schneider, Christian Frings

**Affiliations:** 1 Department of Cognitive Psychology, University of Trier, 54286 Trier, Germany; 2 Department of Computer Science, Trier University of Applied Sciences, Schneidershof, 54293 Trier, Germany

**Keywords:** Neuroscience, Cognitive neuroscience

## Abstract

Optimal mental workload plays a key role in driving performance. Thus, driver-assisting systems that automatically adapt to a drivers current mental workload via brain–computer interfacing might greatly contribute to traffic safety. To design economic brain computer interfaces that do not compromise driver comfort, it is necessary to identify brain areas that are most sensitive to mental workload changes. In this study, we used functional near-infrared spectroscopy and subjective ratings to measure mental workload in two virtual driving environments with distinct demands. We found that demanding city environments induced both higher subjective workload ratings as well as higher bilateral middle frontal gyrus activation than less demanding country environments. A further analysis with higher spatial resolution revealed a center of activation in the right anterior dorsolateral prefrontal cortex. The area is highly involved in spatial working memory processing. Thus, a main component of drivers’ mental workload in complex surroundings might stem from the fact that large amounts of spatial information about the course of the road as well as other road users has to constantly be upheld, processed and updated. We propose that the right middle frontal gyrus might be a suitable region for the application of powerful small-area brain computer interfaces.

## Introduction

Each year approximately 1.35 million people die because of road traffic accidents while another 20–50 million suffer from non-fatal injuries^1^. The vast majority of these accidents are caused by human error^2–4^. Many of these errors occur as a result of excessive demands due to complex traffic situations. To address this issue, more and more driver-assisting systems like advanced cruise and electronic stability control as well as lane and distance-keeping features have been included in cars over the last decades. Already in today’s cars, advanced driver-assisting systems allow to drive for hours without manual interference. In future conditionally automated cars will even allow the driver to ignore the traffic completely during automation and prompt the driver for takeover only in certain situations. It has however, been argued that both too high and too low levels of workload lead to a decline in driving performance^5–8^. Thus, until complete automation is achieved in driving, driver-assisting systems that automatically adept to the drivers’ current mental workload might provide the best improvements regarding traffic safety.

Mental workload has been defined as the portion of processing capacity and resources of an individual that a given task demands^9,10^. For example, in respect to estimate mental workload in driving, it is not sufficient to track the difficulty of the driving environment via indicators like acceleration, deceleration and steering wheel movement. The reason for this is that specific driving maneuvers may challenge the resources of individual drivers in a different manner depending on factors like driving experience, fatigue and present distractions. Thus, mental workload is typically measured with several questionnaires like the NASA TLX^11^ or the ISA^12^. However, especially during difficult tasks it is often not feasible to ask operators directly for their current workload because simultaneously having to make such estimations would impair task performance^13^. To bypass subjective ratings, researchers have tried to find objective correlates of mental workload. Besides unspecific peripheral physiological correlates like heart rate, heart rate variability, blood pressure, respiration, eye blinks and skin conductivity^14^ researchers have tried to pinpoint neuronal structures and processes directly contributing to the processing of demanding tasks.

Thus, there have been attempts to measure the neural correlates of mental workload in driving (-like) tasks with EEG^15–17^ and fMRI^18,19^. However, both fMRI and EEG are quite restrictive in regards to interference caused by participant’s movements and surrounding sources, which makes it hard to implement naturalistic settings in studies that make use of these techniques.

A relatively new method to measure cortical activity is functional near-infrared spectroscopy (fNIRS). fNIRS allows the indirect measurement of neuronal activity via, optically detected changes in oxygenated hemoglobin [oxyHB] and deoxygenated hemoglobin [deoxyHB] concentrations. Both a rise in concentration of oxygenated blood and a decline in concentration of deoxygenated blood can be regarded as the result of neuronal activity. In a laboratory setting, fNIRS-studies have mainly reported two regions that show workload-related activity changes in the n-back task, the ventrolateral prefrontal cortex (VLPFC)^20,21^ and the dorsolateral prefrontal cortex (DLPFC)^21–23^. fNIRS however does not suffer from the same restrictions as fMRI and EEG regarding movement or environmental interferences and thus as well has gained more and more popularity in driving research. In the last decade amongst others, the neuronal correlates of specific driving maneuvers^24–28^, drowsiness and fatigue^29–38^ habituation^39^ and frustration during driving^40^ have been examined with fNIRS. Further, several fNIRS studies have examined the neural correlates of mental workload during different driving operations. These studies in accord with laboratory paradigms have most prominently (though not exclusively) reported a rise of prefrontal activity with rising workload. This link has been reported for several isolated driving maneuvers (lateral prefrontal cortex^41^), secondary tasks during driving (DLPFC, inferior frontal gyrus/ IFG^42,43^), narrow vs. wide rode driving (DLPFC^42^) and driving with differing amounts of automation^44–46^.

To build driver-assisting systems that successfully adapt to the general demand imposed on a driver, rather than transient correlates of single driving maneuvers, it would be useful to find correlates of the general workload imposed on a driver by their environment. So far, not much research has been done in this field. One notable exception is a study by Foy and Chapman^47^ who designed a course comprising four different track types (arterial A-roads, city center multi-lane routes, suburban roads and dual carriageway) and found that prefrontal cortex activity significantly differed between all track types and rose with the mental demand each track type imposed. This study however did not further examine, which prefrontal structures underlie the reported changes. Additionally a rather small sample size of two course segments per track type was used, putting the generalizability of the results into question.

### The present study

In the future, online measurements of neural activity during driving might become a useful tool to gain insight into the driver’s cognitive state. Such information, gathered with fNIRS or similar devices, could be used to automatically engage and disengage driver-assisting systems as required. As a necessary prerequisite however, we need to gain more insight into the neural correlates of driving-related cognitive processes. The goal of this study was to identify specific prefrontal structures activated by mental workload imposed on a driver by their surroundings (i.e. independent of specific driving maneuvers). To this end, we designed two sets of experimental tracks. Within each set, the courses were heterogeneous in regards to routing and thus all required different driving maneuvers. However, routes were designed to have a homogeneous difficulty level within each set. More specifically, we designed a set of country courses, which required standard driving maneuvers like accelerating, breaking, steering and making turns, and a set of city courses, which required the same basic driving maneuvers but additionally required much more stimulus processing, presumably more attention, as well as the regular updating of conceived action plans due to the more complex scenario. Additionally to these experimental tracks, we designed a straight transition track, serving as a conjunction in between each two consecutive experimental track and as a baseline for the change in neural activity in the experimental conditions. Following previous driving research as well as laboratory findings, we measured middle frontal gyrus (MFG) activity with fNIRS during driving. In a first analysis, like previous research, we comprised data in larger regions of interest (ROI), i.e. the left anterior DLPFC (laDLPFC), the left posterior DLPFC (lpDLPFC), the right anterior DLPFC (raDLPFC) and the right posterior DLPFC (rpDLPFC). Additionally (and contrary to most previous research), we also analyzed neural activity separately for each of the 18 channels of our optode mounting. This approach allows us to draw more precise conclusions about specific brain regions involved in driving. Firstly, it allows us to draw comparisons to high resolution neural laboratory research and in turn allows insight into specific processes of action control involved in driving. Secondly, identifying spatially confined brain areas with high sensitivity to mental workload in driving might aid in the development of economical, single-channel BCIs that could be used to control adaptive driver assisting systems in the future.

We hypothesized that both conceived mental workload, as well as MFG activity would be higher during city than during country tracks.

## Results

### Behavioral results

The medium duration it took participants to finish city courses was 80.05 s (SD = 20.50 s). The medium duration it took participants to finish country courses was 63.12 s (SD = 9.35 s). The medium duration it took participants to finish the transition track was 27.76 s (SD = 4.04 s).

**Figure 1:**
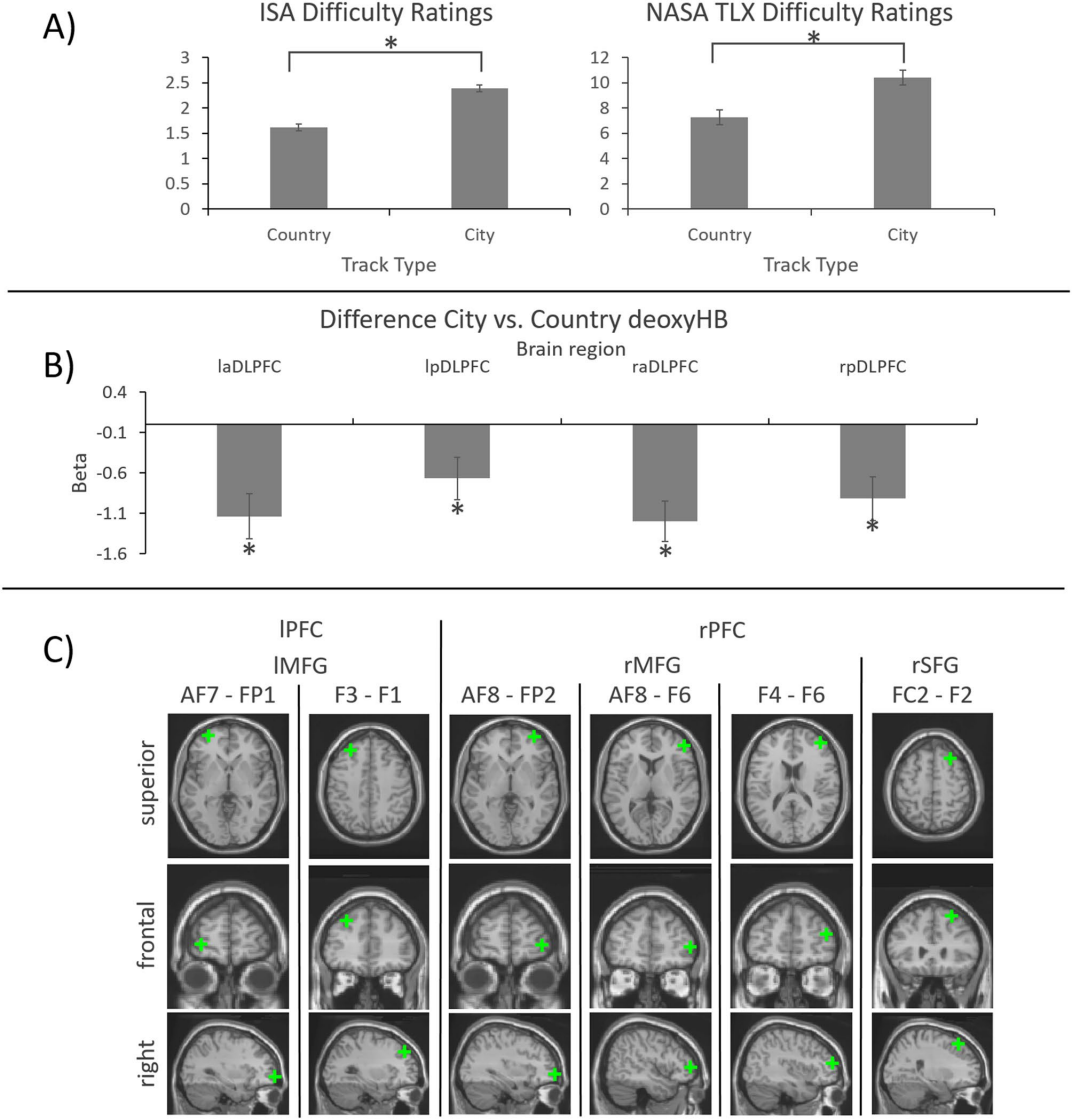
(**A**) ISA and NASA TLX workload ratings. Error bars indicate within-subject design confidence intervals after Morey^61^. Significant differences (*p* < 0.05) are marked with a *. (**B**) β-contrasts for city and country courses in deoxygenated blood for all regions of interest. Significant differences (pFDR corrected *p* < 0.05) are marked with a *. (**C**) Single channel effect localizations. Brain images were created with the WFU Pickatlas, 3.05^62^.

Repeated measurement t-tests revealed significant differences in workload ratings between country and city tracks for both the ISA *t*(21) = 10.75, *p* < 0.001, *d*_*z*_ = 2.32 (mean country = 1.62, SD = 0.36; mean city = 2.39, SD = 0.47) and the NASA TLX *t*(21) = 5.17, *p* < 0.001, *d*_*z*_ = 1.09 (mean country = 7.26, SD = 2.01; mean city = 10.41, SD = 3.17). See Fig. 1A for behavioral results.

### Neuronal results

All four regions of interest (ROI) showed significant differences between conditions in [deoxyHB] indicating higher activity during city courses than during country courses. No ROI showed significant differences between conditions in [oxyHB]. See Table 1 and Fig. 1B for ROI-wise hemodynamic results (see Supplementary Table S1 for the analysis of potential lateralization effects).

**Table 1:** Hemodynamic results depicting all significant contrasts between conditions in oxygenated and deoxygenated hemoglobin*.*

ROI	HB-type	β (SE)^5^	t	p
laDLPFC^1^	Deoxy	− 1.14 (0.28)	− 4.13	< 0.001
lpDLPFC^2^	Deoxy	− 0.67 (0.26)	− 2.6	0.03
raDLPFC^3^	Deoxy	− 1.2 (0.25)	− 4.76	< 0.001
rpDLPFC^4^	Deoxy	− 0.92 (0.27)	− 3.42	< 0.001

^1^Left anterior DLPFC.

^2^Left posterior DLPFC.

^3^Right anterior DLPFC.

^4^Right posterior DLPFC.

^5^All contrast β are depicted with standard errors. All p reflect pFDR corrected significances.

For channel-wise comparisons, 6 channels showed significant differences in [deoxyHB] between conditions. All 6 channels showed higher activation during city courses than during country courses. Activated structures included left MFG (lMFG, 2 channels), right MFG (rMFG, 3 channels) and right SFG (rMFG, 1 channel). No channel-wise differences between conditions were found in [oxyHB]. See Table 2 for significant channel-wise hemodynamic results and Supplementary Table S2 for full channel-wise hemodynamic results. See Fig. 1C for effect locations.

**Table 2:** Hemodynamic results depicting all significant contrasts between conditions in oxygenated and deoxygenated hemoglobin*.*

Brain region	Channel/	HB-type	β (SE)^2^	t	p
	MNI X, Y, Z^1^				
lMFG	AF7–FP1	Deoxy	− 1.53 (0.43)	− 3.53	0.01
	− 33, 59, − 2				
	F3–F1	Deoxy	− 1.33 (0.39)	− 3.38	0.01
	− 31, 39, 41				
rMFG	AF8–FP2	Deoxy	− 1.88 (0.37)	− 5.15	< 0.001
	34, 59, − 2				
	AF8–F6	Deoxy	− 1.38 (0.33)	− 4.2	< 0.001
	48, 46, 5				
	F4–F6	Deoxy	− 1.44 (0.47)	− 3.07	0.03
	40, 50, 16				
rSFG	FC2–F2	Deoxy	− 1.33 (0.45)	− 2.95	0.03
	24, 26, 55				

^1^MNI-coordinates reflect the respective central voxel of the found effects. ^2^All contrast β are depicted with standard errors. All p reflect pFDR corrected significances.

## Discussion

In this study we used fNIRS to examine the frontal hemodynamics associated with different levels of mental workload during simulated driving. To this end, we designed two sets of courses. Both country and city courses required basic driving maneuvers (as accelerating, breaking, steering etc.). However, city courses due to more complex surroundings additionally required more attention and stimulus processing, as well as the regular updating of conceived action plans. Consequently, perceived mental workload, as measured by both the ISA^12^ and the NASA-TLX^11^, was substantially higher during city than during country courses. On a neural level, this effect was reflected by higher activity in all four examined ROIs as indicated by a greater decline in deoxyHB concentrations during city than during country courses in these regions. This finding mirrors the results of previous studies that found a relation between mental workload and prefrontal activity in a range of different driving scenarios like different levels of automated driving^44–46,48^, single driving maneuvers^41^ and longer courses^47^. To gain more insight into the spatial structure of the activation, we conducted a single channel analysis. Channel-wise analysis of neural data offers a significantly higher spatial resolution of neural activation patterns compared to the analysis of larger ROIs. The downside of this detailed analysis is that each result is based on less aggregated data and thus is more susceptible to the influence of artifacts and suffers from a worse signal-to-noise ratio. Nonetheless our analysis revealed six significant effects. Again, all effects indicated higher activity during city compared to country courses and solely presented in deoxyHB. A reason for this could be that fNIRS measurements of prefrontal oxyHB activity (in contrast to deoxyHB activity) are susceptible to systemic artifacts^49^, a problem that might be exacerbated by the motion heavy environment of simulated driving.

Effects were found in three underlying structures, the rMFG the lMFG and the rSFG. Most prior research to (simulated) driving did not engage in channel-wise analysis and thus no comparisons can be drawn. The finding does however coincides with fNIRS^23^ and fMRI^50,51^ laboratory findings regarding spatial working memory, which also most strongly activates rMFG/ rDLPFC regions. It stands to reason that spatial working memory plays an important role in maneuvering complex driving scenarios because drivers have to be aware of and integrate a multitude of fix and moving parts to derive operating action plans.

### Implications for research and application

With modern fNIRS systems, it is easy to measure neural activity over large areas of the skull for research purposes. Using such large-area optode mountings, several brain computer interfacing studies have achieved promising classification accuracies^37,48^. However, future practical application will most likely have to make due with much more limited mountings as to not compromise driver comfort. While driving is a complex tasks, whereby many different brain regions work in accord^24,25,27,28,52^, it is vital to identify those brain regions most sensitive to changes in workload, to maximize the efficiency of brain computer interfaces. We argue that especially the rostral part of the rMFG might be a promising candidate to monitor mental workload during driving. In this region, we found three adjacent channels (AF8—FP2, AF8—F6 and F4—F6) that showed significant differences in activation related to track difficulty. Conceptually it makes sense that the rMFG is especially sensitive to high mental workload in driving. As argued above, the rMFG is closely related to spatial working memory^51^, which in turn is essential in successfully maneuvering difficult driving situations. To our knowledge, only two studies have explicitly examined a potential link between spatial working memory and driving related processes^53,54^. While in a study by Morris et al.^53^ verbal and spatial working memory load did not differentially influence driving performance, Gugerty^54^ found a link between spatial working memory load and attentional processes in driving. Future research should explore the link between mental workload in driving and spatial working memory load using neural imaging. A potential gain from this is twofold. Firstly, should a link between active neural processes during driving and spatial memory processing be established, spatial working memory paradigms could be used to economically train and test machine learning algorithms, before running elaborate field experiments. Secondly, if spatial working memory load indeed constitutes the main component of mental workload during driving, neural research could help in the development of adaptable visual guiding systems.

Beyond this, to further develop and successfully implement assisting systems that adapt to a drivers current needs, we generally have to develop a better understanding of the specific neural processes that underlie driving and how they are strained by environmental demands. While this study provides some preliminary results in this regard, further research is certainly necessary confirm and expand the presented results.

### Conclusion

We used fNIRS to examine the neuro hemodynamic correlates of mental workload during simulated driving. In accordance to previous research, we found that prefrontal activation rose with workload. Beyond previous research, we determined the rostral part of the rMFG as a potential center of workload related activity. We propose that the rMFG is essential in maneuvering complex-driving scenarios in part, because it upholds and integrated spatial information of the environment.

## Methods

### Participants

**Figure 2:**
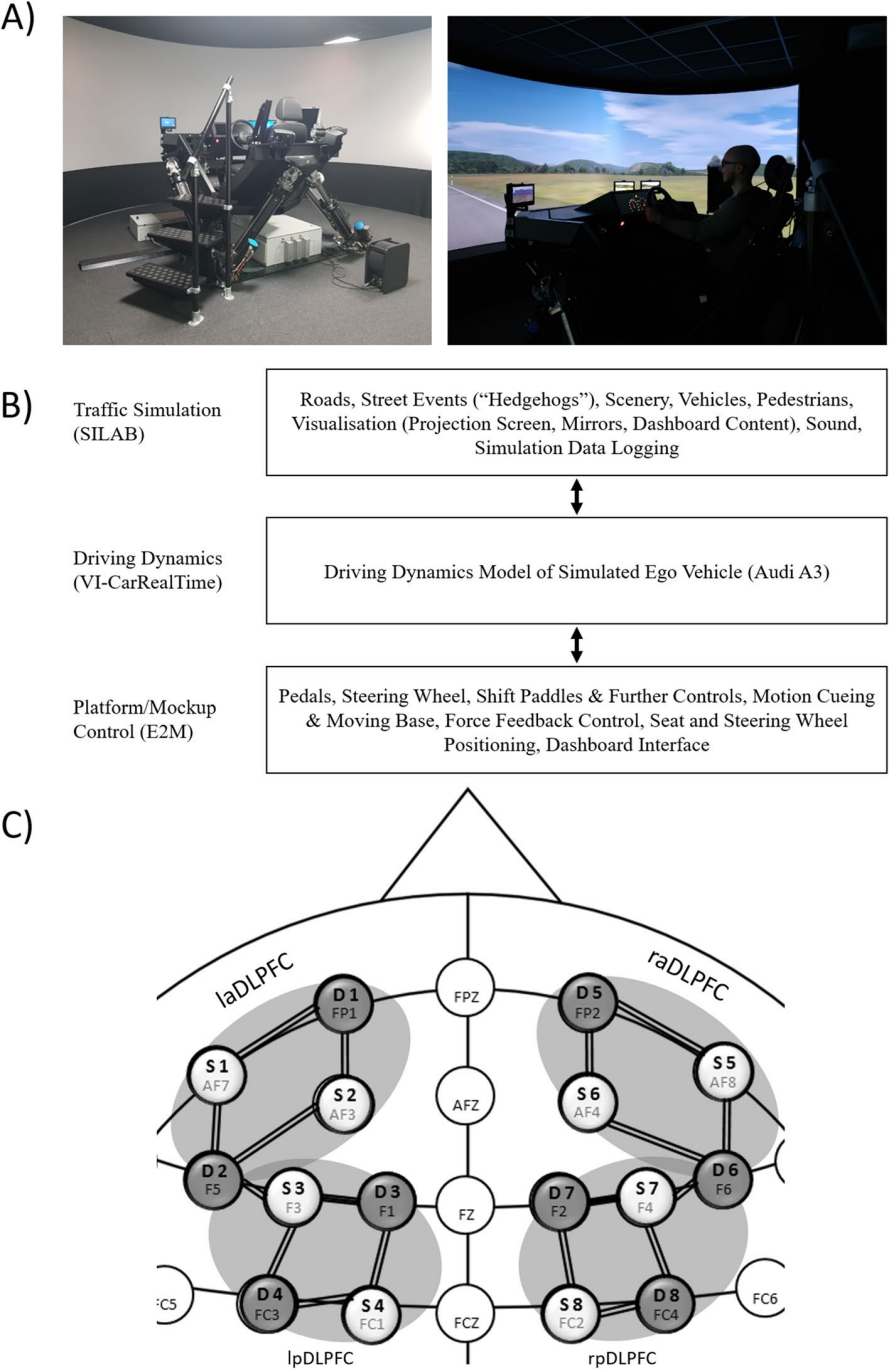
(**A**) FaSiMo driving simulator, left: hexapod in middle position before start up, right: in operation, showing projection on cylindrical screen, mirror displays and dashboard. (**B**) Logical layers of driving simulator stack. (**C**) fNIRS Montage. AF3, AF4, AF7, AF8, F3, F4, FC1 and FC2 were chosen as source positions. FP1, FP2, F1, F2, F5, F6, FC3 and FC4 were chosen as detector positions. These positions where chosen to allow for an optimal coverage of the MFG and resulted in eighteen channels. For additional analysis the channels were pooled into four regions of interest, left anterior DLPFC, left posterior DLPFC, right anterior DLPFC, right posterior DLPFC (laDLPFC: AF7–FP1, AF7–F5, AF3–FP1, AF3–F5; lpDLPFC: F3–F5, F3–F1, F3–FC3, FC1–F1, FC1–FC3; raDLPFC: AF8–FP2, AF8–F6, AF4–FP2, AF4–F6; rpDLPFC: F4–F6, F4–F2, F4–FC4, FC2–F2, FC2–FC4).

Twenty-four participants completed the experiment (several more participated but had to abort the experiment early due to extensive simulator sickness). Two of these participants had to be excluded because of technical problems with the neuronal recoding. The final sample consisted of 22 participants (12 female, median age = 22 years, mean age = 22.23 years with a range of 19–31 years and a standard deviation of 2.72 years). All participants had a valid driver’s license for automobile, stated normal or corrected-to-normal vision and no participant stated any history of neurological disease or predisposition for motion or simulator sickness. Participants gave written informed consent to participation as well as publication of anonymized data before examination and received course credit for their participation. Additionally, the participant shown in Fig. 2A gave written informed consent for publication of an identifying image in an (online) open-access publication. The study was conducted in accordance with the Declaration of Helsinki. Furthermore, the local ethical review committee at the University of Trier evaluated and approved the study.

### Design

The study had a one-factorial (track-type: country route, city route) within subject design.

### Material

The dynamic driving simulator (FaSiMo) of Trier University of Applied Sciences was used to conduct the study (see Fig. 2A). The moving base provides six degrees of freedom (6-DOF), with system performance values as shown in Table 3.

**Table 3:** Driving simulator FaSiMo, system performance values of moving platform*.*

DOF^1^	Position (single)	Position (non-single)	Velocity	Acceleration
Longitudinal	± 300 mm	− 370/+ 360 mm	600 mm/s	11 m/s^2^
Lateral	± 300 mm	± 380 mm	600 mm/s	11 m/s^2^
Vertical	± 260 mm	± 260 mm	500 mm/s	13 m/s^2^
Roll	± 19°	± 23°	40°/s	500°/s^2^
Pitch	− 20°/+ 21°	− 23°/+ 26°	40°/s	500°/s^2^
Yaw	± 20°	± 22°	40°/s	700°/s^2^

^1^Degrees of freedom.

The visual system comprises a 210° cylindrical projection screen, and three displays serving as rear view, and side mirrors, respectively. The cockpit provides a force feedback steering wheel, a driver seat (both electrically adjustable), a three-point seat belt, a digital display as dashboard, controls for headlights and blinkers, shift paddles, accelerator, and brake pedal. The audio system provides a 3D sound model with 5 satellite speakers plus a subwoofer based on dolby-digital 5.1. The control software stack comprises three logical layers: (a) traffic simulation, (b) driving dynamics, and (c) platform/mockup control, as shown in Fig. 2B.

Only adaptations and additions to the upper layer (SILAB) were necessary to facilitate the FaSiMo driving simulator for the conducted study. Besides the actual experimental tracks (see below), a coupling of the SILAB Software with the fNIRS system was developed, to guarantee a time synchronous logging of measured fNIRS data with waypoints. SILAB provides the possibility of street events ("Hedgehogs"), which can be placed at arbitrary points within lanes of simulated streets. Whenever the Ego Vehicle passes over these points, the associated event is triggered. Students of Trier University of Applied Sciences programmed an addition to SILAB to transmit the occurrence of designated "Hedgehogs" via an Arduino Board to the Trigger Input Interface of the used fNIRS System. Thereby, the fNIRS data records contain automatically inserted markers showing when these "Hedgehogs" were "killed".

To get used to the simulator environment and reduce the occurrence of simulator sickness, the participants had to absolve a set of three familiarization courses (provided by WIVW GmbH with the SILAB Software) before the actual experiments. The first course was a straight road, designed to test basic driving maneuvers like accelerating, breaking and swerving about. The second course comprised two sections. The first section prompted emergency breaking at 100 and 120 km/h. The second section prompted double lane changes to evade pylons at 30 and 50 km/h. The third course was a heavily frequented city route including several everyday traffic events. Further, 25 experimental tracks divided into three track types were constructed for/with SILAB (by WIVW GmbH, used Version was SILAB 5.1). These were 12 simple country courses with low traffic volume and without any scripted events, a straight transition track without traffic and a 50 km/h speed limit and 12 city courses with medium to high traffic volume, sidewalks populated with pedestrians and several every day traffic situations. These every day traffic situations included right of way situations, changing traffic lights, pedestrians at crosswalks (either crossing or standing indecisively at the roadside), a traffic circle, waiting school busses (with and without hazard lights), roadworks narrowing the street, a play street with a ball bouncing on the street, cars pulling out of a parking space right ahead of the driver, a motorcycle overtaking the driver, police cars with blue light at a crossing and a police car checking a vehicle at the roadside.

Two common workload measures, the ISA^12^ and the NASA RTLX^11^ were used to capture subjective workload during city and country courses. For the ISA participants were instructed to indicate subjective task-related workload on a scale from 1 to 5 (underutilized, relaxed, comfortable busy pace, high, excessive) using hand gestures (holding up one, two, three, four, five fingers). The ratings were later averaged for all courses of each track type. For the NASA RTLX, task related workload ratings regarding five dimensions (mental effort, physical effort, time pressure, performance, fatigue, frustration) on scales from 1 (minor) to 20 (high, inverted for performance) were averaged for each track type.

### Procedure

After arrival, participants were informed about the general procedure and potential risks of the experiment, filled out the biographic questionnaire and signed consent. Participants then got an introduction regarding the operation of the driving simulator and relevant safety measures in the surroundings of the driver’s cabin. Subsequently they were seated in the simulator and drove the three familiarization courses.

After familiarization, participants were prepared for the fNIRS measurement and got instructions for the experimental courses. During the experiment, participants alternatingly drove country and city courses always divided by the transition track. They were instructed to give ISA difficulty-ratings for the prior city or country course during each transition track.

After the experimental courses, participants left the simulator and the fNIRS cap was removed. Then participants gave overall NASA RTLX ratings for both city and country courses and finally received course credit.

### fNIRS measurement

Hemodynamic changes were recorded with an eight source, eight detector, portable, time-multiplexed, two wavelengths NIRSport (NIRx Medical Technologies LLC, USA) fNIRS device. Optodes were fixed in a standard 10–10 NIRScaps (NIRx Medical Technologies LLC, USA). The placement of fNIRS sources and detectors was chosen utilizing fNIRS Optodes' Location Decider (fOLDv2.2)^55^. fOLD is a Matlab (MathsWorks, USA) based toolbox which computes optimal optode placement in the 10–10 system in regards of covering specific brain areas. For optimal coverage of the MFG AF3, AF4, AF7, AF8, F3, F4, FC1 and FC2 were computed as source positions and FP1, FP2, F1, F2, F5, F6, FC3 and FC4 were computed as detector positions. This resulted in eighteen different channels fourteen of which most likely recorded the MFG while the remaining four most likely recorded the SFG (Fig. 2C depicts the fNIRS Montage, see Supplementary Table S3 for all channel positions). Signals were recorded with NIRStar (NIRx Medical Technologies LLC, USA) recording software with a frequency of 7.81 Hz.

### fNIRS data preprocessing and analysis

NIRS Brain AnalyzIR Toolbox^56^ was used to preprocess and analyze neuro hemodynamic data. For preprocessing, raw voltage data was transformed into light-intensity data and subsequently used to calculate the relative concentration of oxygenated and deoxygenated hemoglobin via Beer–Lambert-Law^57^.Finally, to remove low-frequency characteristics and outliers, a wavelet-filter^58^ was applied. Preprocessed data was then entered into a two-level general linear model (GLM). The first level analysis included four predictors and was conducted for each subject separately. Two predictors coded city and country routes two additional predictors coded intermission tracks and driving mistakes. All predictors were derived from triggers set during the experiment. While triggers for city, country and intermission tracks were automatically set when participants crossed specific waypoints at the beginning of each course, triggers for driving mistakes were manually set by the experimenters. GLM predictors were generated by convolving each event with the canonical hemodynamic response function (HRF). To adapt modeling for individual differences in onset and dispersion of HRF we included the first and second temporal derivative of each prediction term. We corrected for serially autocorrelated errors as well as artifacts induced by systemic physiology and motion with a prewhitening algorithm (AR-IRLS^59^). The predictors for intermission tracks and driving mistakes were excluded from second level analysis, the beta values obtained for city and country courses for each subject were entered into a weighted mixed effects model estimating a fixed intercept for each experimental condition and a random intercept for each subject to best fit the overall data. Betas for each condition were compared for each channel via t-contrasts. Additionally we built four greater regions of interest (as described in^56^), laDLPFC (AF7–FP1, AF7–F5, AF3–FP1, AF3–F5), lpDLPFC (F3–F5, F3–F1, F3–FC3, FC1–F1, FC1–FC3), raDLPFC (AF8–FP2, AF8–F6, AF4–FP2, AF4–F6), rpDLPFC (F4–F6, F4–F2, F4–FC4, FC2–F2, FC2–FC4, see Fig. 2C). To account for alpha inflation due to multiple comparisons p values were corrected applying positive false discovery rate (FDR^60^). Only contrasts that yielded corrected *p* < 0.05 were regarded as statistically significant.

## Supplementary Information

Supplementary Information

## Data Availability

Our data is publicly available via PsychArchives (10.23668/psycharchives.4423).

## Code availability

Our data is publicly available via PsychArchives (10.23668/psycharchives.4424).
